# Comparison of RNA expression profiles on generations of *Porphyra yezoensis *(Rhodophyta), based on suppression subtractive hybridization (SSH)

**DOI:** 10.1186/1756-0500-4-428

**Published:** 2011-10-20

**Authors:** Songdong Shen, Gaochuan Zhang, Yanyan Li, Li Wang, Pu Xu, Lefei Yi

**Affiliations:** 1 Department of Cell Biology, School of Biology and Basic Medical Sciences, Soochow University, Suzhou City, Jiangsu Province, 215123, P. R. China; 2 Department of Biology and Food Engineer, Changshu Institute of Technology, Suzhou City, Jiangsu Province, 215500, P. R. China; 3 Marine Biotechnology Key Construction Laboratory, Huaihai Institute of Technology, Lianyungang City, Jiangsu Province, 226001, P. R. China

## Abstract

**Background:**

*Porphyra yezoensis *Ueda is one of the most important edible seaweed, with a dimorphic life cycle which consists of gametophyte as macroscopical blade and sporophyte as microscopic filamentous. Conspicuous differences exist in the two generations, such as morphology, cell structure, biochemistry, physiology, and so on. The developmental process of *Porphyra yezoensis *has been studied thoroughly, but the mechanism is still ambiguous and few studies on genetic expression have been carried out.

In this study, the suppression subtractive hybridization (SSH) method conducted to generate large-scale expressed sequence tags (EST) is designed to identify gene candidates related to the morphological and physiological differences between the gametophytic and sporophytic generations of *Porphyra yezoensis *Ueda.

**Findings:**

Each 300 clones of sporophyte and gametophyte cells were dipped onto the membrane for hybridization. The result of dot-blot suggested there were 222 positive clones in gametophyte library and 236 positive clones in sporophyte library. 383 positive clones of strongest signals had been sequenced, and 191 EST sequences of gametophyte and 192 of sporophyte were obtained.

A total of 196 genes were obtained, within which 104 genes were identified from the gametophyte and 92 from the sporophyte. Thirty-nine genes of the gametophyte and 62 genes of the sporophyte showed sequence similarity to those genes with known or putative functions which were classified according to their putative biological roles and molecular functions. The GO annotation showed about 58% of the cellular component of sporophyte and gametophyte cells were mainly located in cytoplasm and nucleus. The special genes were located in Golgi apparatus, and high expression in plastid, ribosome and endoplasmic reticulum. The main biological functions of gametophyte cells contributed to DNA repair/replication, carbohydrate metabolism, transport and transcription, especially in response to heat and oxidative stress. The sporophyte cell expresses more genes in transcription, transport, carbohydrate metabolism, particularly in signal transduction, DNA and protein modification, protein and nucleotide metabolism. Four genes are expressed on both gametophyte and sporophyte cells and eighteen genes have not been annotated.

**Conclusion:**

According to the information of GO annotation, the gametophyte tends to growth and self- protection while the sporophyte tends to be more active in development. Interpretation of the differentially expressed genes revealed new insights into the molecular processes of the generation alternation of *Porphyra yezoensis*. Further investigation are needed due to insufficiency of functional genes research and indeterminancy of the functions of many sequences.

## Background

*Porphyra yezoensis *is one of the most important edible seaweeds commercially cultivated mainly in China, Japan and Korea. It has a dimorphic life cycle which consists of macroscopical haploid foliose blade phase and microscopic diploid filamentous conchocelis phase [1]. Conspicuous differences exist in the two generations, such as morphology, cell structure, biochemistry, physiology, and so on. Seed-stock material for *Porphyra *cultivation consists of carpospores (also called zygotespores) [2] spread into oyster shells in the spring, conchocelis germinated and grow in the shells at the onset of conchospores release in the autumn. The conchospores settle on net twine, cultivate in the sea water and develop into blade thalli mainly in winter and early spring. In a mature thallus of *Porphyra yezoensis*, four kinds of cells are developed. From the basal to the top, there are rhizoid cells, vegetative cells, male gametes and female gamete [3].

The developmental process of *Porphyra yezoensis *has been studied thoroughly, but the mechanism is still ambiguous. Very little of the genetic expression has been noticed. What is the process and essential happened in the period from vegetative cells to format sexual cells? What is the difference between the male gametes and female gametes? What is the difference between the foliose blade phase and conchocelis phase?

Nikaido et al (2000) first constructed the normalized and size-selected cDNA libraries of leafy gametophyte *Porphyra yezoensis *and generated a total of 10,154 expressed sequence tags (EST) [4]. Only 934 EST groups were classified based on the annotations of their homologous protein entries in the public databases. The reason for this feature could be that fewer sequences from red and green algae have been registered in the public DNA databases compared to those from higher plants. Asamizu et al (2003) compared the expressed sequence tags (EST) differences between the gametophytic and sporophytic generations of *Porphyra yezoensis *Ueda [5]. Among the 10,625 EST groups, only 1013 (22.5%) groups were classified as ESTs that commonly occurred in both generations, whereas a large proportion of EST groups were identified as being unique to either the gametophyte (43.1%) or the sporophyte (34.3%). A statistical significance test revealed 89 and 112 highly expressed gene candidates in the gametophyte and the sporophyte respectively.

Kakinuma et al (2006) have constructed subtracted cDNA libraries enriched for differentially expressed transcripts in vegetative and reproductive thalli of *Porphyra*, and randomly selected 1,152 cDNAs from each subtracted library [6]. BLAST analysis showed that the cDNAs represented 63 and 59 unique clones for the vegetative and reproductive subtracted libraries respectively. Interestingly, some of the cDNAs isolated from the reproductive subtracted library were homologous to genes encoding protein kinases, GTP-binding protein, and heat shock proteins involved in signal transduction and the molecular chaperon system.

The SSH method is designed to selectively amplify differentially expressed transcripts while suppressing the amplification of abundant transcripts, thus eliminating the need to separate single- and double-stranded molecules. In addition, SSH normalizes target transcripts to approximately equal abundance [7,8].

To identify gene candidates related to the morphological and physiological differences between the gametophytic and sporophytic phases of *Porphyra yezoensis*, suppression subtractive hybridization (SSH) method is conducted to generate large-scale expressed sequence tag (EST). In this study, the carpospores instead of the filamental conchocelis were chosen as sporophyte material. The vegetative (gametophyte) and carpospores (sporophyte) cells live in the same blade of thallus so that the difference between the samples could be neglected.

## Findings

### Suppression subtractive hybridization (SSH)

#### Extraction of RNA

The plant RNA Extraction Reagent was adopted to extract the total RNA from two kinds of *Porphyra yezoensis *cells. Due to the thickness of *Porphyra yezoensis *frond, it contained such large amount of polysaccharose that ordinary RNA Extraction Reagent can not extract the RNA in high quality and the separation of carpospores and blade vegetative cells of *P. yezoensis *were difficult, so it was harder to get much experiment material. For all the reasons, we got the cDNA under the condition of trace total RNA by choosing the SMARTTM PCR cDNA Synthesis kit, it can enrich the Poly A+RNA selectively and reduce the product of cDNA according to the ribosome RNA. Beside these, it can also avoid the cumbersome process of Poly A+RNA enrichment and the samples losses and contamination problems. The value of OD_260_/OD_280 _which estimated by UV spectrophotometer is 1.82. It suggested that there is nearly no protein, phenol, and polysaccharose contamination and no degradation exist through 1% agarose gel electrophoresis. The results suggested that the purity and integrity of total RNA meet the requirement of the SSH experiment.

#### Detection of ligation reaction efficiency

The ligation reaction efficiency of front adaptor would affect not only the result of SSH but also the success ratio of cDNA library. The cDNA mixed with adaptor 1and 2R were respectively reacted by Nest PCR primer1 and PCR primer 2R. According to the result of electrophoresis, the bands are larger than 500 bp and these sequences ligated with the adaptor1 and adaptor 2R also had a good efficiency.

#### Detection of subtractive hybridization efficiency

The subtractive hybridization efficiency of related gene's cDNA was tested by comparing the abundance differences between their subtractive hybridization and without subtractive hybridization.

#### Construction of Subtracted cDNA Library

The reciprocal carpospores and blade vegetative cells were to be tester and driver to construct the forward and reverse Subtracted cDNA library which contained 1000 clones by SSH. Each subtracted cDNA was ligated to Target vector (TOYOBO) and transformed into the component cells (*E. coli *DH5α strain). Picking one clone randomly and using Nest Primer as primer to run the colony PCR to detect the situation of gene inserted into library showed that the size of the products was mainly concentrated on 300-1000 bp which suggested the insert efficiency was nearly 98%.

#### Dot-blot

From the result of dot-blot, the signal of forward subtractive cDNA clone hybridization probe was apparently higher than that of the reverse subtractive one. These clones were the specific expression gene or enhance expression gene in organization. The signal intensity which was similar to the two hybridization probes was thought to be background while no hybridization with two probes was thought to be the lowest abundance cDNA or false positive recombinants. The positive clones were selected by analyzing the image results by optiquant software.

In the experiment, each 300 clones were dipped onto the membrane for hybridization of carpospores and blade vegetative cells. The result of dot-blot suggested that there were 458 positive clones, the positive rate was 76.3% within which 222 positive clones in blade vegetative cells library and 236 positive clones in carpospore library.

### Sequence Analyses

There 383 successes after 408 positive clones of strongest signals were sequenced so that 191 EST sequences of vegetative cells and 192 ESTs of carpospores were obtained. The EST sequences of corresponding clones were trimmed by removing the vector sequences and fuzzy sequences from the former sequences of bases and got 360 in all. All sequences were submitted to the GenBank databases. The assigned GenBank Accession Numbers of leafy carpospores are HS572581- HS572762, and those of leafy vegetative cells are HS572763- HS572942.

### Bioinformatics analysis

The cleaned unigenes/non-redundant sequences were assemblied with more than 20,000 EST sequences deposited in GenBank. Total 196 genes with 104 from vegetative cells and 92 from carpospores were obtained,. Some genes were represented by multiple ESTs as shown in the Additional file 1and Additional file 2 that included the contigs with more than 170 ESTs. Database searches and similarity analyses of cDNA nucleotide sequences were carried out with the BLASTN and BLASTX programs against public nucleotide, EST, and protein databases [9,10]. Thirty-nine of the vegetative cells and 62 of the carpospores that showed sequence similarities to known or putative function proteins were classified according to their putative biological roles and molecular functions (Table 1).

**Table 1:** The list of gene name annotated by Gene Ontology based on the homologous protein entries in the public databases

Gene symbol	Full Name	vegetative cells	carpospores
NeoTet	pNeo4 NeoTet		√
fglh1	lysyl hydroxylase 1		√
response regulator	response regulator		√
sensor histidine kinase	sensor histidine kinase		√
Peb1	response regulator Rr1 genes		√
rr1	response regulator Rr1		√
	sigma-54 DNA-binding response regulator	√	
	sigma 54 modulation protein	√	
RPL41	ribosomal protein L41	√	√
RPL27A	ribosomal protein L27A	√	
RPL30	ribosomal protein L30		√
RPL23a	ribosomal protein L23a	√	
RPL9	ribosomal protein L9	√	√
RPS3	ribosomal protein S3		√
RPS11	ribosomal protein S11	√	√
EEF1A1	eukaryotic translation elongation factor 1 alpha 1		√
RSIP	ribosomal subunit interface protein	√	
GTP1/OBG family	translation-associated GTPase	√	
RNA helicase	DEAD-box ATP dependent DNA helicase	√	
Snf2 family protein	Snf2 family protein-related Helicase	√	
tRNA synthase	tRNA pseudouridine synthase	√	
RecQ	ATP-dependent DNA helicase RecQ	√	
MutT	putative mutator protein	√	
NUDIX hydrolase	MutT/nudix family protein	√	
uvrA	excinuclease ABC, subunit A	√	
integrase	Transposase from transposon Tn916		√
	DNA-directed RNA polymerase subunit		√
recF	recombination protein F		√
RadA	DNA repair protein		√
hsdM	type I restriction enzyme M protein		√
RE	restriction endonuclease		√
GidA	tRNA uridine 5-carboxymethylaminomethyl modification enzyme	√	
GidA	glucose-inhibited division protein A	√	
fruA/fruB	frutan hydrolase (fruA), FruB (fruB)	√	
ek435	cyplasin S		√
hsp68	heat shock protein 68	√	
hrcA	repressor of heat shock gene expression HrcA	√	
dnaK	molecular chaperone DnaK	√	
grpE	molecular chaperone	√	
PUM1	pumilio 1	√	
CYTH2	cytohesin 2		√
CBX7	protein binding		√
PSCD2	pleckstrin homology, Sec7 and coiled domains 2		√
APOBEC3A			√
Acac	nucleolin-related protein, transcript variant		√
INHBA	inhibin beta A		√
RBM9	RNA binding motif protein 9		√
SLC25A5L	solute carrier family 25		√
PAX1	paired box gene 1		√
E1BP1			√
g6pd	glucose-6-phosphate-1-dehydrogenase		√
thiol peroxidase	thiol peroxidase	√	
SOD1	superoxide dismutase 1		√
BTG1	B-cell translocation gene 1, anti-proliferative		√
ORFA			√
ctc			√
aab	aromatic amino acid biosynthetic gene cluster		√
Ndfip1	Nedd4 family interacting protein 1	√	
RGS7	regulator of G-protein signalling 7	√	
LAPTM4A	lysosomal-associated protein transmembrane 4α	√	
ABC transporter	putative osmoprotectant ABC transporter	√	√
nitrite transporter	nitrite transporter	√	
p76	Transmembrane 9 superfamily member 2 precursor		√
IBP	iron-binding protein genes		√
eep			√
EF2015	minor head protein	√	
HAD	HAD superfamily hydrolase	√	
methyltransferase	putative methyltransferase	√	
carboxylesterase	carboxylesterase		√
Gfo/Idh/MocA	family oxidoreductase		√
SEPHCHC synthase	carboxylic acid synthase		√
PTS system	phosphotransferase system		√
SAG0894 family	CRISPR associated protein		√
SecY	preprotein translocase subunit SecY	√	
signal peptidase I	signal peptidase I		√
clpC	stress response-related Clp protease	√	
NDUFA9	NADH dehydrogenase (ubiquinone) 1 alpha		√
ATP citrate synthase	ATP citrate synthase		√
ATP citrate lyase	ATP citrate lyase		√
atpA/B/C/D/E/F/G/H	H+ ATPase subunits	√	
COX4I1	cytochrome c oxidase subunit IV isoform 1		√
pykF	pyruvate kinase		√
Rmet_0948	ubiquinol oxidase, subunit II		√
FH	fumarate hydratase	√	
ADPGlc Ppase	Glucose-1-phosphate adenylyltransferase		√
glycerol dehydrogenase	glycerol dehydrogenase		√
dapB	dihydrodipicolinate reductase		√
cmk	Cytidine monophosphate kinase		√
arcABCRD	arginine deiminase operon		√
argR1/argR2	arginine repressors		√
pI			√
h1			√
NGLY1	N-glycanase 1		√
prs	phosphoribosyl pyrophosphate synthetase		√
ldh	L-lactate dehydrogenase		√
gcaD	UDP-N-acetylglucosamine pyrophosphorylase		√
ghfp	glycosy hydrolase family protein	√	
EF2591	glyoxalase family protein		√
adk	adenylate kinase(AK)	√	
YgfK	pyridine nucleotide-disulfide family	√	
ApbE	thiamin biosynthesis ApbE	√	
IPP isomerase	phosphomevalonate kinase	√	
Gelsolin precursor	Gelsolin precursor		√
IVNS1ABP	influenza virus NS1A binding protein		√
holin	holin	√	
psbO1	oxygen-evolving enhancer 1 precursor	√	
PBP 1B	membrane carboxypeptidase		√
MDRP	multidrug resistance protein		√
MFSP	major facilitator superfamily protein		√

**Figure 1:**
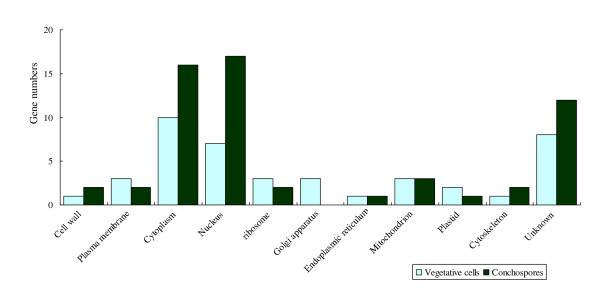
**The GO annotations of Cellular Component**.

**Figure 2:**
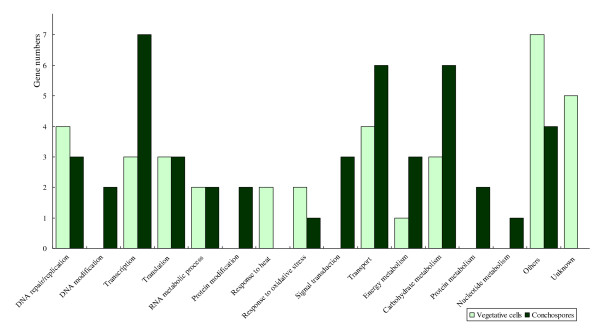
**The GO annotations of Biological Process and Molecular Function**.

The GO annotation showed the cellular component of carpospores were mainly located in cytoplasm and nucleus, reaching to 58%; while vegetative cells also mainly located in cytoplasm and nucleus, especially located in Golgi apparatus, and abundance in plastid, ribosome and endoplasmic reticulum (Figure 1). The main biological functions of vegetative cells were related to DNA repair/replication, carbohydrate metabolism, transport and transcription, particularly in response to heat and oxidative stress. The carpospores expressed more transcripts in transcription, transport, carbohydrate metabolism, especially in signal transduction, DNA and protein modification, protein and nucleotide metabolism (Figure 2). According to the information of GO annotation, the vegetative cells tend to growth and self- protection, while the carpospores tend to more active in development. Four genes namely ribosomal protein L41, 50S ribosomal protein L9, ribosomal protein S11 and ATP-binding protein are expressed on both vegetative cells and carpospores. Eighteen genes have not been annotated. Because in this study functional genes are still not enough and the functions of many sequences are not clear, so further investigations are needed.

## Discussion

The morphological and physiological differences between the leafy gametophyte and the filamentous sporophyte in the life cycle of *Porphyra *species are so great that many studies have been performed. Gametophyte- and sporophyte-specific cDNAs of *Porphyra purpurea *which encode proteins such as elongation factors, serine protease-like proteins, polysaccharide-binding proteins, and lipoxygenases had been isolated by differential screening and subtraction of phase-specific cDNA libraries (Liu et al., 1994, 1996) [11-15].

For single direct partial sequencing of anonymous cDNA clones of *Porphyra purpurea *performed by Liu et al was not available in obtaining enough genetic information. Lee et al. constructed the EST library of *P. yezoensis *thullus [16]. Among 190 ESTs generated, 81 sequence tags carried partial cDNA with significant amino acid sequence similarity to known genes, but the other 109 clones were not homologous to previously identified genes. At the same time, EST analysis has been performed to identify candidate genes related to the morphological and physiological differences between the gametophytic and sporophytic generations, and 20779 cDNAs have been identified [4,5]. However, because these are mainly new genes, without homologous to known ones, the genes which regulate the life history process of *Porphyra *thalli remain poorly understood.

Gametogenesis of *Porphyra *thalli was induced by changing the photoperiod and water temperature. Kakinuma et al. (2006) have constructed subtracted cDNA libraries enriched for differentially expressed transcripts in vegetative and reproductive thalli, and randomly selected 1,152 cDNAs from each subtracted library [17]. Results of the dot blot analyses used for identification of differentially expressed cDNAs and BLAST analysis of nucleotide and deduced amino acid sequences showed that the cDNAs represented 63 and 59 unique clones for the vegetative and reproductive. Some of the cDNAs isolated from the reproductive subtracted library were homologous to genes encoding protein kinases, GTP-binding protein, and heat shock proteins involved in signal transduction and the molecular chaperon system. However, it is possible that changes in expression of these genes may only be a response to the change in temperature and photoperiod and are unrelated to gametogenesis.

To identify genes involved in the *Porphyra yezoensis *asexual sporulation, Kitade, et al. (2008) compared the gene expression profiles derived from gametophytes and sporophytes using cDNA macroarray, which includes 4,896 nonredundant expressed sequence tag (EST) groups. Candidate genes were screened by two different macroarray data analyses combined with reverse transcription-PCR (RT-PCR) analysis and Northern analysis. RT-PCR analysis revealed that nine genes were expressed in gametophyte specific manner, and two genes were expressed only in gametophytes that formed archeospores [18].

The cDNA microarray provides a high capacity and credibility system for the investigation of *Porphyra yezoensis *genes expression profile. For example, the different generations of *P. yezoensis *467 functional gene clones were obtained and dotted onto the slides by cDNA microarray which coated by poly-lysine. The analysis of the normalized data showed that 55 genes were excessively expressed in gametophyte generation, 21 of which were similar to the genes of known or putative function; 86 genes were excessively expressed in sporophyte generation, and among them, 24 were similar to the genes of known or putative functions [19].

Compared with previous works on *Porphyra yezoensis *generation related gene expression, we constructed the difference expression libraries by the suppression subtractive hybridization (SSH) method The GO annotation showed that 58% cellular components of carpospores were located in cytoplasm and nucleus; while vegetative cells were mainly located in cytoplasm and nucleus, especially located in Golgi apparatus, and high expression on plastid, ribosome, endoplamic reticulum. In the thallus of vegetative growth, photosynthesis and carbohydrate assimilation, RNA transcription and protein translation, and material transport are the main biological processes. According to the cultivation manner of *P. yezoensis *in China, the thalli are aerial exposure on every six hours during low tide. The extreme tolerance to desiccation of high intertidal *P. yezoensis *is demonstrated and blades survive a loss of up to 85-95% of their water during the daytime low tide to become crisp sheets. Temperature (winter freezing stress at high latitudes and heat stress in summer) and highlight stress often accompanies the cultivation period. The genes in response to heat and oxidative stress are expressed at large amount.

The carpospores express more genes in transcription, transport, carbohydrate metabolism, particular in signal transduction, DNA and protein modification, protein and nucleotide metabolism. The carpospores tends to be more active in development, rather in growth so that the signal transduction and a series of metabolisms happened in order to get enough energy for releasing and drilling into calcium carbonate shell. The expressed sequence tags of filamentous sporophyte of *Porphyra haitanensis *analysis studied by Pang et al. also found the transport and transcription, responsing to oxidation genes expression, and so on, similar to this research [24].

Subtractive hybridization is the first technique to be widely used for the purpose of identifying differentially expressed genes on a global scale. Advantages of the technique include the ability to isolate genes with no prior knowledge of their sequence or identity and the use of common molecular biology techniques that do not require specialized equipment or analyses. Several limitations of the original protocols, such as requirements of large quantities of RNA and bias toward abundant genes, have been overcome by incorporation of PCR into the suppression subtractive hybridization (SSH) technique. However, SSH remains applicable only on pair-wise treatment comparisons and must be replicated with the tester and driver reversed to identify gene expression changes in both directions. Additionally, subtractive hybridization does not provide a quantitative measure of expression differences and is most efficient at identifying genes that are completely absent, rather than expressed less abundantly, in the driver sample.

Though the suppression subtractive hybridization (SSH) technique can overcome the bias toward abundant genes more effective than subtractive hybridization, there are still four genes obtained in this research, such as ribosomal protein L41(HS), 50S ribosomal protein L9(HS), ribosomal protein S11 (HS572889 and HS572733) and ATP-binding protein (HS572892 and HS572609) which are identified on both vegetative cells and carpospores. These genes are too abundant to remove their mRNAs so that they can be amplified by the subsequent PCR process. This is another limitation of SSH.

Eighteen genes have not been annotated in this study. Due to the insufficient research on functional genes and unclear function of many sequences, further investigations are needed.

## Conclusion

According to the information of GO annotation, the vegetative cells tend to growth and self- protection, while the carpospores tends to be more active in development. Interpretation of the differentially expressed genes revealed new insight into the molecular processes of the generation alternation of *Porphyra yezoensis*. Due to the insufficient research on functional genes and unclear function of many sequences, further investigations are needed.

## Methods

### Suppression subtractive hybridization (SSH)

#### The separation of *Porphyra yezoensis *cells

The mature blades of *Porphyra yezoensis *were collected from the culture nets in the sea located in the Dongtai City, Jiangsu Province. Carpospore and blade vegetative cells of *Porphyra yezoensis *were separated under the dissecting microscope to ensure its correctness. After that, the separated cells were collected in the freezing tubes which had pretreated with DEPC solution separately and were preserved in liquid nitrogen for future use.

#### The extraction of total RNA and synthesis of full-length cDNA

About 0.1 g separated cells that preserved in liquid nitrogen were put into the pre-cold mortar with liquid nitrogen. Then the cells were grounded into powder and were added 1 ml plant RNA Extraction Reagent to extract the total RNA. There was no degradation observed through 1% agarose gel. The RNA purity and quantities were measured by UV spectrophotometer method. The extracted RNAs were stored at -80°C for future use. The synthesis of cDNA was carried out according to the Super SMART PCR cDNA Synthesis Kit.

#### SSH process

According to the protocol of Clontech PCR-Select cDNA Subtraction Kit, the adaptor was connected and the ligation efficiency was estimated after the products were digested by Rna I (or *Rsa*I) enzyme and purified. Carpospores were used as tester and blade vegetative cells were as driver, they were hybridized twice at 68°C for 8 h and 16 h respectively. Then the specific amplification of forward subtractive cDNA was gotten by twice inhibition PCR. At the same time, the reverse subtractive cDNA was performed with the tester and driver exchanged

#### The construction of subtractive cDNA library of differential expression between generations

The differentially expressed cDNAs between generations were ligated into the Target Vector (TOYOBO), then transformed into the component *E. coli *DH5α and the white-blue plaque selection were used to get the positive clones.

#### The detection of dot- blot

Fifty nanogram carpospore and 50 ng blade vegetative cells of Porphyra yezoensis were treated with *Rsa *I enzyme. The random primed labeling method was adapted on the double strands DNA and the radionuclide ^α-32^P dATP was used to label the probes.

The 480 clones were picked randomly from library, and the amplification of colony PCR was constructed by using nested primer. The products larger than 800 bp were dipped on the nitrocellulose membrane (double membrane prepared), and then the dot-blot was carried on. Fifty nanogram forward and reverse subtractive cDNA groups were marked as probe to hybrid with the two repeated copies membranes of the same cDNA library.

#### EST processing

The two groups of EST sequences were cleaned with the CAP3 software.

Low quality sequences and vector sequences were removed from each EST clone by using this application. The cleaned EST sequences were used to find overlap assembly of contiguous sequences. Cap3 was used to identify overlaps between sequences and assemble sequence fragments into a larger sequence [20]. Samples that could be joined together were assembled into contiguous sequences "contigs". Those sequences from a cluster allowing the establishment of a consensus sequence were included in a contig. In this process, we defined singlets as clustered sequences that could not be included in a consensus sequence and singletons as sequences that were not grouped in a cluster. The unigenes were then the sum of singletons, singlets, and contigs.

#### EST comparative analysis and functional assignment

We performed Homologous search by Blastn tool with the unique sequences (including assembled contigs and singletons). A local implementation of BLAST server was used to search against the NCBI's non-redundant nuclear sequence database. We set up a cut off value and discarded hits with an E-value < 10-5. Parameters were as follow: Max target sequences: 10 (Descriptions:10, Alignments:10); Expect threshold: 0.01; Wordsize: 11; Match Score: 2; Mismatch Score: -3; Gap Open Penalty Score: 5; Gap Extension Penalty Score: 2.

The homology searches were made again with the program Blastx, with a threshold e-value of 1e-20. Because the conserved of amino acids sequence is higher than that of nuclear sequence, the ESTs sequences can be translated into amino acid sequences and searched the homology with Blastx for those ESTs without obvious results in Blastn. Parameters were as follow: Max target sequences: 10 (Descriptions:10, Alignments:10); Expect threshold: 0.01; Wordsize: 3; Matrix: BLOSUM62; Gap Open Penalty Score: 11; Gap Extension Penalty Score: 1.

To assign putative functions to the unique sequences, we employed blast2go to extract the GO hierarchical terms of their homologous genes from the protein databases [21]. Meanwhile, we also mapped the unique sequences to metabolic pathways in accordance with the KEGG [22]. Enzyme commission (EC) numbers [23] were obtained and used to putatively map unique sequences to specific biochemical pathways.

## Competing interests

The authors declare that they have no competing interests.

## Authors' contributions

SS and PX initiated the study. YL and LW performed the RNA isolation, suppression subtractive hybridization (SSH) and sequencing. SS, GZ and LY designed the framework for data analysis and interpretation of data.

SS drafted the original manuscript. All authors have participated in the final editing and have read and approved the final manuscript.

All authors read and approved the final manuscript.

## Supplementary Material

Additional file 1 **The identification of non-redundent singlets and contigs of vegetative cells from SSH library**. The file contains SSH clone ID, cDNA insert size, putative identify, E-value, cellular component, biological process and molecular function annotation from the vegetative cells. Click here for file

Additional file 2 **The identification of non-redundent singlets and contigs of carpospores from SSH library**. The file contains SSH clone ID, cDNA insert size, putative identify, E-value, cellular component, biological process and molecular function annotation from the carpospores. Click here for file
